# What’s in the Gift? Towards a Molecular Dissection of Nuptial Feeding in a Cricket

**DOI:** 10.1371/journal.pone.0140191

**Published:** 2015-10-06

**Authors:** Yannick Pauchet, Natalie Wielsch, Paul A. Wilkinson, Scott K. Sakaluk, Aleš Svatoš, Richard H. ffrench-Constant, John Hunt, David G. Heckel

**Affiliations:** 1 Entomology, Max Planck Institute for Chemical Ecology, Jena, Germany; 2 Mass spectrometry, Max Planck Institute for Chemical Ecology, Jena, Germany; 3 Centre for Ecology and Conservation, College of Life and Environmental Sciences, University of Exeter, Cornwall Campus, Penryn, United Kingdom; 4 Behavior, Ecology, Evolution & Systematics Section, School of Biological Sciences, Illinois State University, Normal, IL, United States of America; CNRS, FRANCE

## Abstract

Nuptial gifts produced by males and transferred to females during copulation are common in insects. Yet, their precise composition and subsequent physiological effects on the female recipient remain unresolved. Male decorated crickets *Gryllodes sigillatus* transfer a spermatophore to the female during copulation that is composed of an edible gift, the spermatophylax, and the ampulla that contains the ejaculate. After transfer of the spermatophore, the female detaches the spermatophylax and starts to eat it while sperm from the ampulla are evacuated into the female reproductive tract. When the female has finished consuming the spermatophylax, she detaches the ampulla and terminates sperm transfer. Hence, one simple function of the spermatophylax is to ensure complete sperm transfer by distracting the female from prematurely removing the ampulla. However, the majority of orally active components of the spermatophylax itself and their subsequent effects on female behavior have not been identified. Here, we report the first analysis of the proteome of the *G*. *sigillatus* spermatophylax and the transcriptome of the male accessory glands that make these proteins. The accessory gland transcriptome was assembled into 17,691 transcripts whilst about 30 proteins were detected within the mature spermatophylax itself. Of these 30 proteins, 18 were encoded by accessory gland encoded messages. Most spermatophylax proteins show no similarity to proteins with known biological functions and are therefore largely novel. A spermatophylax protein shows similarity to protease inhibitors suggesting that it may protect the biologically active components from digestion within the gut of the female recipient. Another protein shares similarity with previously characterized insect polypeptide growth factors suggesting that it may play a role in altering female reproductive physiology concurrent with fertilization. Characterization of the spermatophylax proteome provides the first step in identifying the genes encoding these proteins in males and in understanding their biological functions in the female recipient.

## Background

Nuptial gifts occur in several insect taxa and come in various forms, such as food items collected by males, various body secretions, body parts or even the male’s entire body, and are exchanged during courtship or copulation [1, 2]. Nuptial gifts play crucial roles in both pre- and postcopulatory sexual selection and have been re-examined in the context of sexual conflict [1, 3, 4]. Their extreme diversity prompted Lewis and colleagues [5] to propose a two-part classification of nuptial gifts. The first is based on their source: ‘endogenous’ gifts are produced or sequestered by donors (typically males), whereas ‘exogenous’ gifts are captured or collected from the surrounding environment. The second takes into account the way nuptial gifts are received by recipients (typically females) and they can be classified as (i) oral gifts eaten and absorbed through the digestive system, (ii) genital gifts absorbed through the reproductive tract or (iii) transdermal gifts injected directly through the insect’s cuticle [5].

In the decorated cricket, *Gryllodes sigillatus*, the male produces and transfers a bipartite spermatophore to the female during copulation. The spermatophore comprises (i) an oral gift in the form of a large gelatinous portion (the spermatophylax) that the female removes and feeds on following spermatophore transfer (and thus during sperm transfer); and (ii) the ampulla containing the ejaculate, which the female detaches and consumes immediately after having entirely consumed (or discarded) the spermatophylax, thereby terminating sperm transfer. There is a direct correlation between the time needed for a female to entirely consume a spermatophylax and the time required for complete transfer of sperm from the ampulla to the female’s sperm storage organ (termed spermatheca) [6]. Hence, consumption of the spermatophylax by the female functions to ensure that sperm transfer is completed. Conversely, if a female prematurely discards the gift before entirely consuming it, sperm transfer is incomplete, thus reducing the number of offspring sired by the male that transferred the spermatophylax. The spermatophylax must therefore be both appealing to the female and sufficiently large to delay her subsequent removal and consumption of the sperm-containing ampulla.

Spermatophylaxes produced by male *G*. *sigillatus*, as well as by various species of bush crickets, are composed of 80–85% water and the rest is mostly protein, peptides and free amino acids, representing up to 90% of the dry weight [7, 8]. Importantly, spermatophylaxes produced by male *G*. *sigillatus* have little or no nutritional value [9]; spermatophylax consumption does not contribute directly to the fitness of the female or the progeny she produces. In *G*. *sigillatus*, free amino acids represent 7% of the dry mass of a spermatophylax. Warwick and colleagues [8] demonstrated that the exact composition of free amino acids found in *G*. *sigillatus* spermatophylaxes acts as a phagostimulant, a result validated by a subsequent multivariate selection analysis of the amino acid composition of the spermatophylax [10, 11].

Although the composition of the free amino acids in the spermatophylax has recently received considerable attention [8, 12], comparatively little is known about its protein composition, the major component of its dry mass. This represents a major deficit in our understanding of spermatophylax structure and function because proteins could, in theory, play an important role in mediating two important evolutionary conflicts between the sexes arising from the provision of the spermatophylax: 1) a sexual conflict over whether the female accepts the gift; in fact, it is not widely appreciated that females often discard the spermatophylax by simply dropping it prior to its complete consumption in approximately 25% of all matings, behavior that is inimical to the fitness interests of the male because females invariably remove the sperm ampulla prematurely in such cases; 2) a sexual conflict over female remating that appears to be mediated, in part, by refractory-inducing substances contained in the spermatophylax In fact, previous work suggests that the spermatophylax contains anti-aphrodisiacal compounds to which female *G*. *sigillatus* have evolved resistance, as evidenced by an increase in refractory periods that ensued when these gifts were fed to females of non-spermatophylax-donating crickets [13]. We therefore investigated the protein composition of *G*. *sigillatus* spermatophylaxes using proteomics. To facilitate protein identification, we coupled proteomics analysis to the transcriptome sequencing of male accessory glands, the tissue producing spermatophylaxes in male *G*. *sigillatus*.

## Methods

### Cricket rearing

*Gryllodes sigillatus* used in this study were descended from 500 adult crickets collected in Las Cruces, New Mexico, in 2001, and used to initiate a laboratory culture maintained at a population size of approximately 5000 crickets. No specific permissions were required to collect *G*. *sigillatus* because these specimens were collected in a non-protected area and this species is not endangered or protected. Crickets, which were allowed to breed panmictically, were housed in 55-L plastic containers in an environmental chamber maintained at 32 ± 1°C on a 16 h:8 h light/dark cycle. Crickets were provisioned with Harlan 2018CM Teklad Certified Global 18% Protein Rodent Diet meal, water provided in 40 mL plastic tissue culture flasks plugged with cotton dental rolls, and egg cartons to provide shelter and to increase the rearing surface area. Moistened peat moss provided in small plastic containers was made available both as an oviposition substrate and as a source of additional water [14].

### Accessory gland cDNA library preparation, sequencing and assembly

Five pairs of accessory glands were dissected from mature *G*. *sigillatus* males, immediately frozen in liquid nitrogen and stored at -80°C prior to RNA extraction. Total RNA was isolated using TRIzol reagent (Invitrogen, Carlsbad, CA, USA) according to the manufacturer’s instructions. Genomic DNA was removed by incubation with DNAse (TURBO DNAse, Ambion, Austin, TX, USA) for 30 min at 37°C. Accessory gland RNA was further purified by the RNeasy MinElute Clean up Kit (Qiagen, Valencia, CA, USA), again, following the manufacturer’s protocol, and then eluted in 20 μl RNA storage solution (Ambion). Full-length, enriched cDNAs were generated from 2 μg total RNA using the SMARTer PCR cDNA synthesis kit (BD Clontech, Mountain View, CA, USA). Reverse transcription was performed with the SMARTScribe Reverse Transcriptase (BD Clontech) for 60 min at 42°C and 90 min at 50°C. In order to prevent overrepresentation of the most common transcripts, the resulting double-stranded cDNAs were normalized using the Kamchatka crab duplex-specific nuclease method [15] of the trimmer-direct cDNA normalization kit (Evrogen, Moscow, Russia). For 454 pyrosequencing, a cDNA aliquot of the accessory gland library was sent to the Advanced Genomics facility at the University of Liverpool (http://www.liv.ac.uk/agf). A single full-plate run of 454 Titanium (Roche Applied Science) was performed using 3 μg of normalized cDNAs processed by the ‘‘shotgun” method. All 454 derived sequences have been submitted to the Short Read Archive (SRA) database at NCBI with accession SRR2136652 (Bioproject ID PRJNA291561). Trimming and assembly of the raw nucleotide sequences was achieved using the ‘*est2assembly*’ pipeline [16]. The resulting contigs of the assembled *G*. *sigillatus* accessory gland transcriptome were subsequently translated in the six possible open reading frames (ORFs) using a custom script, compiled in a protein database and used for the proteomics analyses described below.

### Spermatophylax protein extraction and gel electrophoresis

Spermatophylaxes were collected from mature *G*. *sigillatus* males by gently squeezing their spermatophore pouch, which causes them to extrude the spermatophylax. After collection, spermatophylaxes were immediately snap-frozen in liquid nitrogen, lyophilized and stored at -80°C until use. Ten lyophilized spermatophylaxes were homogenized with a Potter-Elvehjem tissue homogenizer in 2 ml 20 mM Tris-HCl pH 8.0 containing a protease inhibitor cocktail (COMPLETE EDTA-free, Roche). After centrifugation (16,000 x g, 15 min, 4°C), the supernatant was collected in a new tube and filter-sterilized. The whole 2 ml spermatophylax extract was loaded on a 1 ml RESOURCE Q anion exchange chromatography column (GE Healthcare) connected to an Äkta FPLC system (GE Healthcare). After extensive washing of the column, bound proteins were eluted using a 0 to 1 M linear NaCl gradient over 30 column volumes. Eluted proteins were recovered in 1 ml fractions. One hundred microliters of each fraction containing a protein peak at 280 nm were precipitated by 10% trichloroacetic acid, using 0.02% sodium deoxycholate as a co-precipitant; the final pellets were dissolved and boiled in 15 μl SDS-PAGE sample buffer. Samples were then loaded on a Criterion XT gradient 4–12% polyacrylamide SDS-PAGE gel (BioRad) and run for 2 h in XT MES running buffer at a constant voltage of 120 V. The gel was then fixed in 40% (v/v) ethanol, 10% (v/v) acetic acid, for 2 h and stained using Colloidal Coomassie as described by Neuhoff et al. [17]. Finally, gels were scanned using a GS800 densitometer (BioRad) and analyzed using Quantity One software version 4.6.3 (BioRad).

### In-gel digestion and peptide extraction

Protein bands of interest were cut out from the Coomassie-stained gel and put in a microcentrifuge tube. Proteins were reduced in-gel by 10 mM dithiothreitol and alkylated by 55 mM iodoacetamide. Destained, washed, dehydrated gel pieces were rehydrated for 60 min in 12 ng/μl solution of porcine trypsin (Promega) previously dissolved in 25 mM ammonium bicarbonate buffer at 4°C and then digested overnight at 37°C. The tryptic peptides were extracted from gel pieces with 75% acetonitrile/5% formic acid, and the extracts were dried out in a vacuum centrifuge. For LC-MS/MS, analysis samples were reconstituted in 10 μL aqueous 1% formic acid (FA).

### LC-MS/MS analysis

Samples were separated using a nanoAcquity nano UPLC system (Waters, Manchester, UK). A mobile phase of 0.1% aqueous FA was used to concentrate and desalt the sample on a Symmetry C18 trap column (20 x 0.18 mm, 5 μm particle size) at a flow rate of 15 μL per min. Subsequently, peptides were eluted onto a nanoAcquity C18 column (200 mm ×75 μm ID, C18 BEH 130 material, 1.7 μm particle size) using an increasing acetonitrile gradient in 0.1% aqueous formic acid at a flow rate of 0.35 μl per min. Buffers A (0.1% FA) and B (100% MeCN in 0.1% FA) were linearly mixed in a gradient from 1% to 55% phase B over 60 min, increased to 95% B over 5 min, held at 95% B for 5 min and decreased to 1% B over 1 min. The analytical column was immediately re-equilibrated for 9 min. The eluted peptides were transferred to the nanoelectrospray source of a Synapt HDMS tandem mass spectrometer (Waters, Manchester, UK) equipped with a metal-coated nanoelectrospray tip (Picotip, 50 × 0.36 mm, 10 μm internal diameter, New Objective, Woburn, MA, USA). The source temperature was set to 80°C, cone gas flow 20 L/h, and the nanoelectrospray voltage was 3.2 kV. For all measurements, the mass spectrometer was operated in V-mode with a resolution power of at least 10,000 FWHM. All analyses were performed in positive ESI mode. A 650 fmol/μL human Glu-fibrinopeptide B in 0.1% FA/acetonitrile (1:1 v/v) was infused at a flow rate of 0.5 μL per min through the reference Nano-LockSpray source every 30 seconds to compensate for mass shifts in MS and MS/MS fragmentation mode. LC-MS data were collected using data-dependent acquisition. The acquisition cycle consisted of a survey scan covering the range of m/z 400–1500 Da followed by MS/MS fragmentation of the three most intense precursor ions collected at 1 sec intervals in the range of 50–1700 m/z. Dynamic exclusion was applied to minimize multiple fragmentations for the same precursor ions.

### Data processing and protein identification

DDA raw files were collected using MassLynx v4.1 software and processed using ProteinLynx Global Server Browser (PLGS) v2.5 software (Waters, Manchester, UK) under baseline subtraction, smoothing, deisotoping, and lockmass-correction. Processed spectra were interpreted *de novo* to yield peptide sequences. A 0.002 Da mass deviation for *de novo* sequencing was allowed, and sequences with a ladder score (percentage of expected y- and b-ions) exceeding 40 were subjected to sequence-similarity searching using the MS BLAST program [18] installed on an in-house server. MS BLAST searches were carried out against the Insecta subdivision of the GenBank non-redundant protein database, the orthopteran subdivision of InsectaCentral (http://insectacentral.org/) and the *in silico* translated *G*. *sigillatus* accessory gland transcriptome using the following settings: scoring Table, 100; Filter, none; Expect, 100; matrix, PAM30MS; advanced options, no-gap-hspmax100-sort_by_totalscore-span1. Statistical significance of hits was evaluated according to the MS BLAST scoring scheme [18]. Scoring of the significance of peptide matches is not based on *E*- or *p*-values of the individual HSPs (high-scoring segment pairs) but instead on precomputed threshold scores conditional on the number of query peptides and HSP hits.

### Manual curation of cDNAs and full-length sequencing

Contigs corresponding to spermatophylax proteins were retrieved from the *G*. *sigillatus* accessory gland transcriptome, re-assembled one by one using the SeqMan Pro assembler version 10.1.2 (DNASTAR, Madison, WI, USA), and manually curated to correct potential assembly errors. Contigs encoding only a partial open reading frame (ORF) were used to design specific primer pairs to perform 5’- and 3’-rapid amplification of cDNA ends (RACE) PCRs. For these we used the SMARTer RACE cDNA Amplification Kit (BD Clontech) according to the manufacturer’s instructions. All cDNA sequences corresponding to spermatophylax proteins were annotated and submitted to Genbank under accession numbers KT355854 to KT355874.

### Assembly of cricket accessory gland ESTs from public databases

EST datasets from *Allonemobius fasciatus*, *Gryllus firmus* and *Gryllus pennsylvanicus* [19] were retrieved from the dbEST public database (NCBI) as FASTA files. These datasets were then assembled using the SeqMan Pro assembler of the Lasergene software package 10.1.2 (DNASTAR, Madison, WI, USA) with the following program parameters: match size, 50bp; minimum match percentage, 80%; minimum sequence length, 40 bp; gap-length penalty, 0.70 and maximum mismatch end bases, 15. The accessory gland transcriptome from *Teleogryllus oceanicus* [20] was retrieved from the short-read archive (SRA) at NCBI (accession SRX090654) and assembled using CLC Genomics Workbench v6.0.1. First, sequences were trimmed for length and quality with standard settings. Subsequently, they were assembled using the following CLC parameters: nucleotide mismatch cost = 2; insertion = deletion costs = 2; length fraction = 0.3; similarity = 0.9. Any conflicts among the individual bases were resolved by voting for the base with highest frequency. Contigs shorter than 250 bp were removed from the final analysis.

### Sequence alignments

Amino acid sequences were searched for the presence of an amino-terminal signal peptide using the SignalP version 4.1 server [21]. After their signal peptide was removed, amino acid sequences were aligned with MUSCLE (v3.7) configured with default settings and the resulting alignments were inspected and corrected manually when needed before being further edited using Jalview version 2 [22].

## Results and Discussion

### Mapping of the *Gryllodes sigillatus* spermatophylax proteome

**Fig 1:**
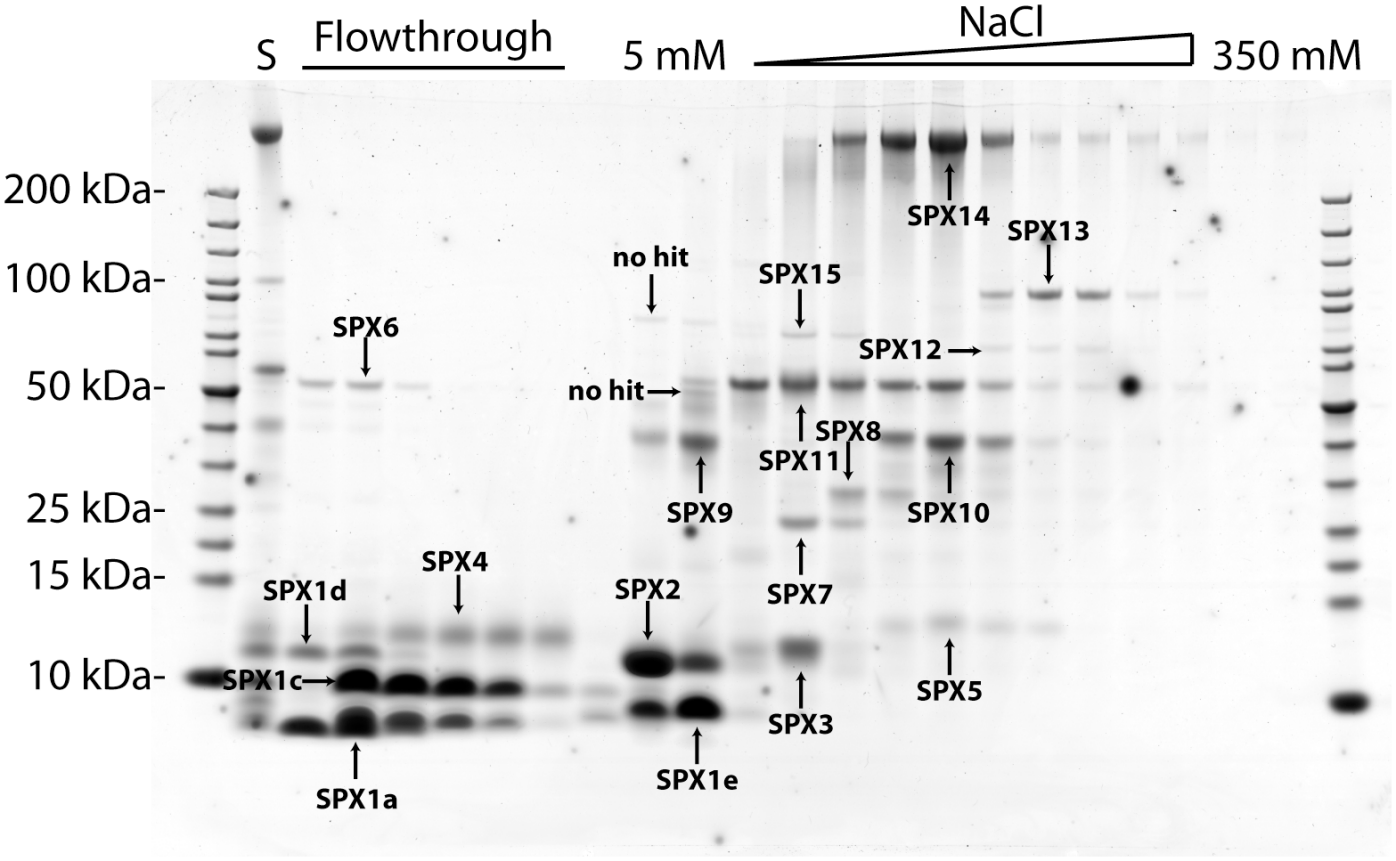
Separation of proteins from male *Gryllodes sigillatus* spermatophylaxes using a two-dimensional proteomics approach. Spermatophylax proteins from *G*. *sigillatus* were separated by anion exchange chromatography (first dimension). Each 1 ml fraction containing a significant amount of protein was resolved on a gradient (4–12%) SDS-PAGE gel (second dimension) and stained with colloidal Coomassie blue. Molecular weight markers in kilodaltons are indicated to the left of the gel. Proteins that bound to the anion exchange column were eluted between 5 and 350 mM NaCl. Protein bands were recovered from the gel and digested with porcine trypsin for further mass spectrometry analyses and protein identification. Proteins positively identified are labelled SPX1 to SPX15. Initial spermatophylax protein extract prior to anion exchange chromatography is labeled ‘S’.

Proteins extracted from lyophilized spermatophylaxes were resolved by SDS-PAGE and stained with colloidal Coomassie blue (Fig 1). The resulting protein profile was surprisingly simple with major protein bands ranging from as low as <10 kDa to as high as >200 kDa, very similar to the spermatophylax protein profile of another orthopteran, the tettigoniid, *Bolivarius siculus* [23]. To increase the separation and resolution of the spermatophylax proteins, we separated them by anion exchange chromatography and considered this step as the first dimension of a two-dimensional approach, similar to our previous work on *Manduca sexta* and *Phaedon cochleariae* [24, 25]. About half of the proteins did not bind to the anion exchange chromatography column and were recovered in the flowthrough, whereas the other half did bind to the column and proteins were eluted with NaCl concentration ranging from 5 to 350 mM. After resolving the fractions that contained a protein pick (absorbance at 280 nm) by SDS-PAGE, corresponding to the second dimension of a two-dimensional approach, and after staining by colloidal Coomassie blue, a relatively simple pattern of distinct protein bands was revealed (Fig 1). Twenty of these protein bands, corresponding to apparently unique protein products, were recovered and analyzed by mass spectrometry.

As *G*. *sigillatus* is a non-model organism from a genomic point of view, we decided to interpret the tryptic-digest peptides corresponding to the protein bands we collected *de novo* and search these *de novo* peptide predictions using MS-BLAST, a program developed to utilize redundant, degenerate and partly inaccurate peptide sequences in similarity searches of protein databases that may be derived from organisms phylogenetically distant from the study species [18, 26]. First, we searched the *de novo* peptides against the Insecta subdivision of the GenBank non-redundant protein database, but we were unable to identify a single spermatophylax protein (abbreviated SPX in the rest of the text) although this approach has proven to be successful in the past [25, 27]. As a second step, we searched our *de novo* peptide predictions against the subset “Orthoptera” of InsectaCentral (http://insectacentral.org/), a central repository of insect transcriptomes, produced using traditional capillary sequencing (Sanger) or next generation sequencing (NGS). Using this second approach, we managed to confidently identify three SPX proteins having a high degree of similarity with other orthopteran-derived proteins (Table 1), namely a chitinase-like (SPX6), a fasciclin-like (SPX12) and a transferrin-like (SPX15) protein.

**Table 1:** Summary of the identifications obtained searching Orthoptera-derived EST datasets on InsectaCentral.

Name	InsectaCentral accession	Species	MS-BLAST		InsectaCentral annotation
			Peptides^a^	Score^b^	Description
SPX6	IC109027AaApep254	*Laupala kohalensis*	7	394	Chitinase-like
	IC109027AaApep124	*Laupala kohalensis*	4	203	Chitinase-like
SPX12	IC7000AaApep116	*Gryllus firmus*	3	189	Fasciclin-like
SPX15	IC7000AaApep89	*Gryllus firmus*	5	284	Transferrin
	IC109027AaApep139	*Laupala kohalensis*	5	259	Transferrin
	IC109027AaApep175	*Laupala kohalensis*	4	197	Transferrin

^a^Number of peptides matching best hit in MS-BLAST search.

^b^MS-BLAST scoring (please refer to Material and Methods section). Details related to these peptides can be found as supplementary information (S1 File).

Our inability to identify SPX proteins using publicly available protein databases prompted us to generate a *G*. *sigillatus*-specific protein database that we could subsequently use to search our *de novo* peptide predictions. In order to be as specific as possible and to increase the chance of confidently identifying SPX proteins, we decided to generate a transcriptome of the tissue that produces spermatophylaxes in male *G*. *sigillatus*, namely the accessory glands. We assumed that most (if not all) proteins integrated into spermatophylaxes are produced by the male accessory glands, although we cannot exclude the possibility that some proteins may be synthesized elsewhere and transported via the hemolymph to the spermatophylaxes. We generated a normalized cDNA library from accessory glands dissected from sexually active male *G*. *sigillatus*. The resulting transcriptome was obtained by pyrosequencing this cDNA library (454 Titanium) and assembling the resulting sequencing reads. The resulting *G*. *sigillatus* male accessory gland transcriptome was assembled into 15,879 contigs and 1,812 singlets (Table 2) which were then translated *in silico* in the six possible ORFs and compiled into a protein database searchable via MS BLAST [18]. Searching the *de novo* peptide predictions obtained by mass spectrometry and corresponding to the 20 unique protein products present in the spermatophylax protein extract against this newly created protein database allowed us to confidently link the majority (17 out of 20) of these protein products to individual sequences present in the accessory gland transcriptome (Table 3). One of the three unidentified protein products corresponded to SPX15, which was previously identified as a putative transferrin searching an orthopteran-specific protein database on InsectaCentral. We then performed BLAST searches against a non-redundant protein database (NCBI Genbank) in order to annotate the *G*. *sigillatus* SPX proteins we identified using our proteomics data (Table 3); nine out of the 17 identified protein sequences—among these, the highly abundant SPX1s and SPX2—returned no BLAST hits and would thus correspond to novel proteins with no homologs in model insects. Among the eight *G*. *sigillatus* SPX proteins that returned a BLAST hit were a serine protease inhibitor harboring two pacifastin domains, an odorant-binding protein, a chitinase-like protein also annotated as a “wing disc growth factor-like protein”, a serine carboxypeptidase, two carbonic anhydrases, a fasciclin-like protein and a protein with similarity to an accessory gland protein derived from other cricket species (Table 3).

**Table 2:** Summary statistics for the assembly and annotation of the *Gryllodes sigillatus* male accessory gland transcriptome.

*Assembly*	
Total number of reads	1,191,716
Total number of reads after QC^a^	1,103,771
Average read length after QC^a^	358 bp
Total number of contigs	15,879
Total number of singlets	1,812
Average contig size	1,171 bp
*Annotation*	
Contigs with a BLAST hit^b^	57.3%
Contigs with at least one gene ontology (GO) term	48.9%
Contigs with an enzyme code (EC)	9.8%
Contigs with at least one InterPro domain	40%

^a^QC stands for quality control. This step primarily removes reads of poor sequencing quality after base calling. It also removes from reads those sequences that correspond to adapters and are used to construct the normalized cDNA library as well as those added for the sequencing itself.

^b^BLASTx was used to annotate the transcriptome against the non-redundant protein database at NCBI, with an expected value cutoff of 10^−6^.

**Table 3:** Summary of the identifications obtained searching the *G*. *sigillatus* male accessory gland EST dataset.

Name	Status of cDNA	Length ORF (bp)	Length predicted protein (aa)	MS-BLAST		BLASTp vs NCBI nr			
				Peptides^a^	Score^b^	Description^c^	Species^d^	Accession^e^	E-value^f^
SPX1a	Full-length	258	85	4	270	No hit	-	-	-
SPX1c	Full-length	279	92	5	432	No hit	-	-	-
SPX1d	Full-length	276	91	4	294	No hit	-	-	-
SPX1e	Full-length	273	90	4	365	No hit	-	-	-
SPX2	Full-length	294	97	6	466	No hit	-	-	-
SPX3	Full-length	432	143	2	132	Odorant binding protein 2c	*Locusta migratoria*	ACR39390	6.9e-24
SPX4	Full-length	357	118	1	78	Pacifastin	*Culex quinquefasciatus*	EDS34849	9e-16
SPX5	Full-length	363	120	2	139	No hit			
SPX6	Full-length	1299	432	12	1019	Imaginal disc growth factor-like protein	*Mamestra brassicae*	ABC79625	2.8e-146
SPX7	Partial	702	233	10	782	Retinoid inducible serine carboxypeptidase	*Pediculus humanus corporis*	EEB17508	6.6e-35
SPX8	Full-length	726	241	4	202	No hit	-	-	-
SPX9	Full-length	834	277	17	1206	Carbonic anhydrase II	*Aedes aegypti*	EAT37074	1.9e-28
SPX10	Full-length	858	285	10	852	Carbonic anhydrase	*Tolumonas auensis*	WP012728610	10e-25
SPX11	Full-length	1032	343	10	642	No hit	-	-	-
SPX12	Partial	2109	702	7	470	Fasciclin	*Gryllus veletis*	ABG07890	1.8e-174
SPX13	Partial	3039	1013	2	150	No hit	-	-	-
SPX14	Partial	1641	547	9	494	Putative accessory gland protein	*Gryllus pennsylvanicus*	ABG01748	5.5e-06

^a^Number of peptides matching best hit in MS-BLAST search.

^b^MS-BLAST scoring (please refer to Material and Methods section). Details related to these peptides can be found as supplementary information (S2 File).

^c^Result of blastp search using SPX predicted protein as query against NCBI non-redundant (nr) protein database.

^d^Species corresponding to best hit.

^e^NCBI accession number.

^f^ *E*-value of best hit in blastp search against NCBI nr.

### Distribution of SPX protein homologs in other orthopteran accessory gland transcriptomes

Several male accessory gland transcriptomes for species of Orthoptera other than *G*. *sigillatus* are currently publicly available. One of these, derived from the field cricket *Teleogryllus oceanicus*, was generated by 454 pyrosequencing and is comparable to the transcriptome we generated for *G*. *sigillatus* [20]. In addition, low-coverage expressed sequence tag (EST) datasets are also available for three other cricket species, *Allonemobius fasciatus*, *Gryllus firmus* and *Gryllus pennsylvanicus* [19]. We recovered these transcriptome datasets from Genbank (NCBI) and assembled them into contigs and singletons. Importantly, males of these four cricket species do not provide a spermatophylax to females at mating. We used the *G*. *sigillatus* SPX protein sequences as queries to mine these four cricket transcriptomes for the presence of SPX protein homologs (Table 4). Not too surprisingly, we found the highest number of SPX homologs, five in total, in the *T*. *oceanicus* accessory gland transcriptome. We found homologs for the chitinase-like protein (SPX6), the serine carboxypeptidase (SPX7), one of the two carbonic anhydrases (SPX9), the fasciclin-like (SPX12), and for a protein with no homologs in model insects (SPX13). No homologs of the highly abundant SPX1s and SPX2 proteins could be found in any of these four male accessory gland transcriptome datasets.

**Table 4:** Presence/absence of SPX protein orthologs in other male accessory gland transcriptomes from other orthopteran species.

SPX proteins	*Allonemobius fasciatus*		*Gryllus firmus*		*Gryllus pennsylvanicus*		*Teleogryllus oceanicus*	
	Presence	aa identity	Presence	aa identity	Presence	aa identity	Presence	aa identity
SPX1s	No	-	No	-	No	-	No	-
SPX2	No	-	No	-	No	-	No	-
SPX3	No	-	No	-	No	-	No	-
SPX4	No	-	No	-	No	-	No	-
SPX5	No	-	No	-	No	-	No	-
SPX6	No	-	No	-	No	-	Yes	92%
SPX7	No	-	Yes	78%	No	-	Yes	70%
SPX8	No	-	No	-	No	-	No	-
SPX9	No	-	No	-	No	-	Yes	80%
SPX10	No	-	No	-	No	-	No	-
SPX11	No	-	No	-	No	-	No	-
SPX12	Yes	78%	Yes	95%	No	-	Yes	96%
SPX13	No	-	No	-	No	-	Yes	85%
SPX14	No	-	No	-	No	-	No	-

### A novel protein family highly abundant in *G*. *sigillatus* spermatophylaxes

**Fig 2:**
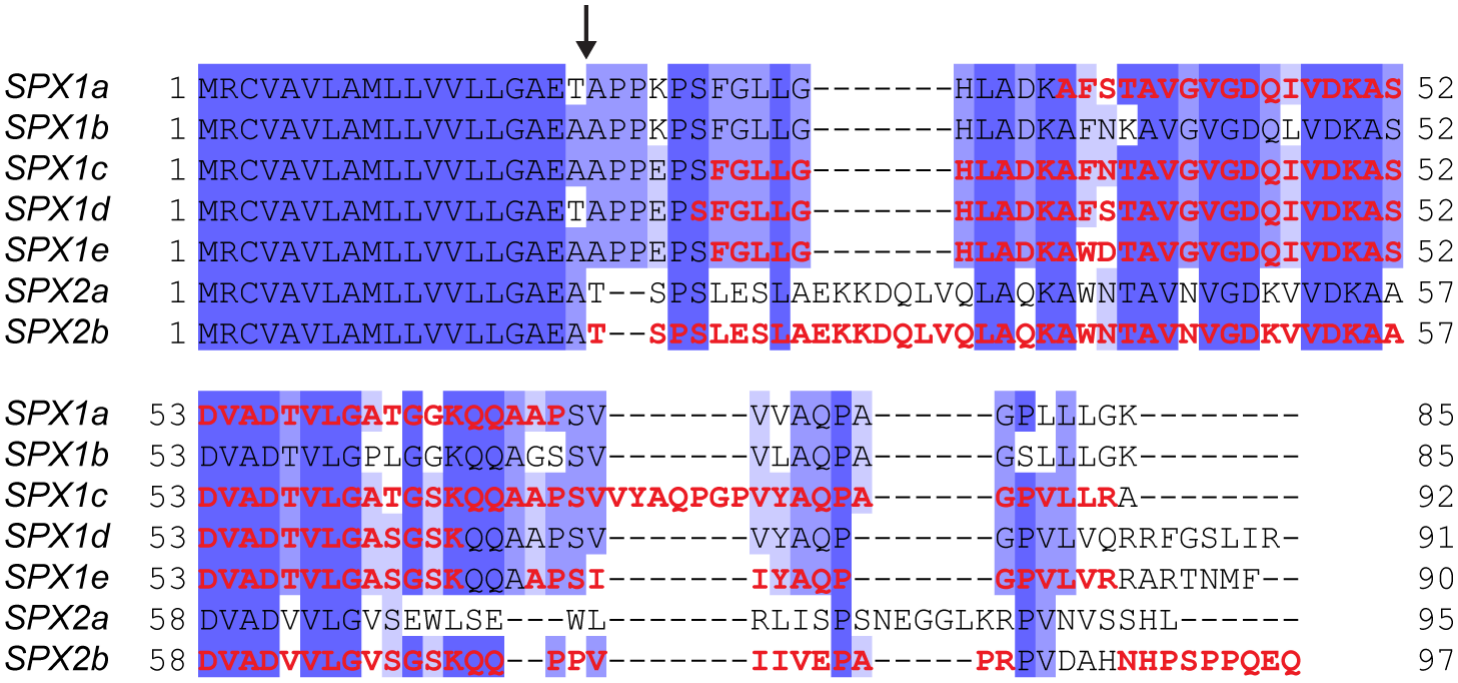
Amino acid alignment of the highly abundant SPX1a-1e and SPX2a and 2b proteins. Protein sequences were aligned using Muscle v3.7 and the degree of amino acid identity of each residue is represented by dark (strictly identical) to light purple shadings (low identity). The cleavage site of the predicted signal peptide determined using SignalP v4.1 is indicated by an arrow. *De novo* sequenced peptides obtained after mass spectrometry and used for the identification of these proteins are indicated in red.

The most abundant proteins present in *G*. *sigillatus* spermatophylaxes (SPX1a, SPX1c to 1e and SPX2b) corresponded to a group of relatively small polypeptides with an apparent molecular weight ranging from well below to slightly above 10 kDa. The amino acid sequences corresponding to these abundant polypeptides were related to each other, shared a high percentage identity, and could easily be aligned (Fig 2). The amino acid sequence corresponding to the signal peptide was found to be completely identical (except for its last amino acid between these five proteins). Due to their high degree of conservation, several of the *de novo* sequenced peptides obtained after mass spectrometry did match to several SPX1 variants; in all cases, however, the exact variant was identifiable due to one or several discriminant peptides (peptides matching to only a single SPX1 variant). Similarly, we found two variants for SPX2 in our accessory gland transcriptome, but from the *de novo* peptides we obtained, there was no ambiguity in the identification of SPX2b (Fig 2). We then performed an alignment of the nucleotide sequences corresponding to the SPX1 and SPX2 variants (S1 Fig). These nucleotide sequences, which could be aligned relatively easily, harbored highly conserved 5’- and 3’-untranslated regions (UTRs). Although SPX1 and SPX2 variants could be clearly distinguished from each other by observing these sequence alignments, our analyses did not provide enough evidence for us to determine whether the SPX1 variants corresponded to different genes or to spliced variants of a single gene. In the case of SPX2, due to the perfect identity of the 5’- and 3’-UTRs between SPX2a and SPX2b, we speculated that these two SPX2 sequences may be spliced variants of a single gene (S1 Fig). We attempted to amplify these genes from genomic DNA using several primer combinations, but, surprisingly, we never managed to get any PCR products. Orthopteran genomes are in general quite large; some of them have been estimated to be up to several gigabases [28]. The large size of orthopteran genomes could be reflected in the size of gene introns and could explain our lack of success in amplifying SPX1 and SPX2 genes by PCR. As already mentioned, no homologs of SPX1 and SPX2 variants could be found using BLAST searches—neither in model insects nor in male accessory gland transcriptomes derived from four cricket species; at this stage of our analyses, we cannot speculate about what the biological function of these novel spermatophylax proteins might be. One advantage of the high degree of identity between SPX1 and SPX2 variants is that a single double-stranded RNA (dsRNA) construct could be sufficient to knock down the expression of these genes simultaneously using RNA interference (RNAi). RNAi is the method of choice for knocking down the expression of target genes in order to analyze their function. Several recent reports indicated that RNAi seems to work well in species of Orthoptera [29–31], and we hope to use this technique in the future to unravel the function of SPX1 and SPX2 variants.

### A serine protease inhibitor located in *G*. *sigillatus* spermatophylaxes

We identified SPX4 as a member of the pacifastin-like family of serine protease inhibitor (Fig 1 and Table 3). Members of the pacifastin family are largely distributed in arthropods, and these polypeptides are generally composed of an amino-terminal signal peptide followed by a variable number of inhibitor domains [32]. SPX4 follows the general structure of pacifastin-like proteins; its precursor is composed of a signal peptide followed by two pacifastin inhibitor domains, each of which is characterized by 6 conserved cysteine residues forming three disulfide bridges (S2 Fig). Based on knowledge acquired from the study of pacifastins derived from the desert locust *Schistocerca gregaria*, we predict that the first pacifastin domain is probably a chymotrypsin inhibitor, based on the presence of a leucine residue at the P1 position. Similarly, we also predict that the second pacifastin domain is probably a trypsin inhibitor, based on the presence of an arginine residue at the P1 position [33, 34]. The P1 residue, located in the so-called canonical peptidase-binding loop P3–P3’, has been shown to be crucial for the binding of, and specificity towards, the target peptidase [33]. If our predictions are true, this would make SPX4 a bi-functional inhibitor capable of blocking chymotrypsin and trypsin activities.

Gwynne [1] proposed several reasons for why oral gifts, such as spermatophylaxes, are unlikely to contain specialized allohormonal chemicals that affect specific target tissues in the female. One of Gwynne’s main arguments is that complex chemicals (including proteins) consumed orally—those that could influence female behavior and fitness—would probably be subjected to enzymatic breakdown in the female’s digestive tract [1]. However, it has been demonstrated that pacifastin-like peptides isolated from the desert locust *S*. *gregaria* are very efficient in inhibiting the locust’s own digestive serine proteinases, even if they are less efficient in inhibiting commercially available mammalian-derived trypsins [34, 35]. Similarly, SPX4 may possess the ability to inhibit *G*. *sigillatus*’ own digestive proteinases—most of which are trypsins [36]—and thus could protect the other SPX proteins from proteolysis after the ingestion of a spermatophylax by a female cricket. If this hypothesis is true, then potential effector proteins present in the spermatophylax could indeed reach their target(s) in the female body with a limited risk of being degraded during their passage through the female’s digestive tract. Thus, access to a female’s physiology via substances ingested during spermatophylax feeding remains a viable mechanism by which males may manipulate female behavior (or reproduction) as an extension of sexual conflict.

### Presence of a potential polypeptide growth factor in spermatophylaxes

**Fig 3:**
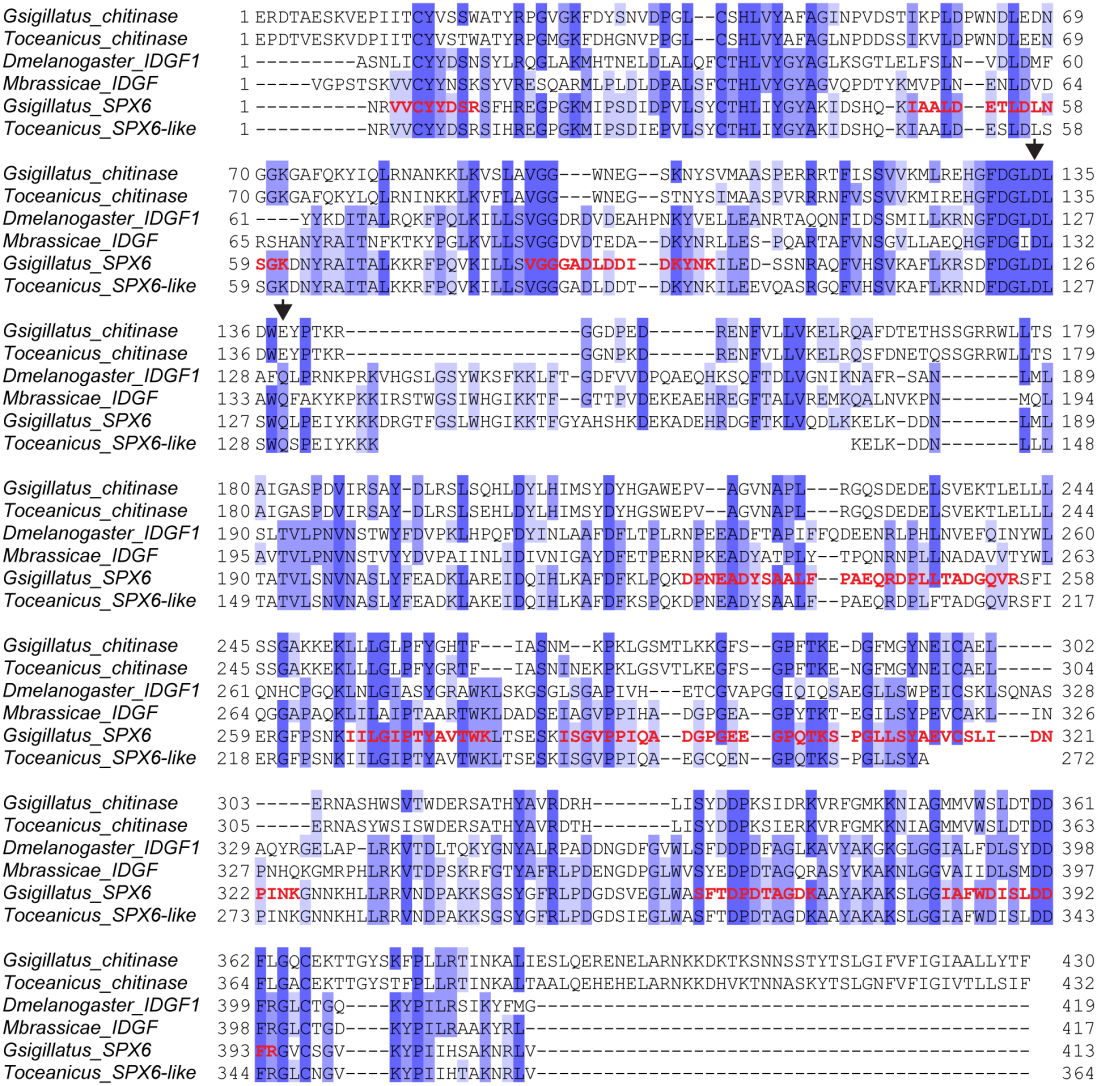
Amino acid alignment of *Gryllodes sigillatus* SPX6 with homologs from other species. *Gryllodes sigillatus* SPX6 sequence was aligned with a true chitinase, a member of GH18 family, found in the accessory gland transcriptome (*Gsigillatus*_chitinase); a true ortholog found in an accessory gland transcriptome of the cricket species *Teleogryllus oceanicus* (*Toceanicus*_SPX6-like); a true ortholog of *Gsigillatus*_chitinase found in an accessory gland transcriptome of *T*. *oceanicus*; and various “imaginal disc growth factors” derived from other insect species (*Dmelanogaster*_IDGF1, *Mbrassicae*_IDGF). Protein sequences were aligned after removing part of the sequence corresponding to the amino-terminal signal peptide using Muscle v3.7, and the degree of amino acid identity of each residue is represented by dark (strictly identical) to light purple shadings (low identity). Empty spaces present in the alignment of *T*. *oceanicus* SPX6-like correspond to region where no sequence information was available. The two conserved catalytic residues found in true chitinases are indicated by arrowheads. The *de novo* sequenced peptides obtained by MS/MS which made it possible to identify *G*. *sigillatus* SPX6 are indicated in red.

SPX6 showed high similarity to chitinase-like proteins member of the glycoside hydrolase family 18 (GH18) (Fig 1 and Table 3). An amino acid alignment between SPX6 and other members of the GH18 family indicated that the amino acid corresponding to the catalytic proton donor, a glutamate residue in active chitinases, was substituted for a glutamine in SPX6, indicating that this protein is most certainly not an active enzyme (Fig 3). The same amino acid substitution of the catalytic proton donor was also observed in a SPX6 ortholog found in the *T*. *oceanicus* transcriptome as well as in two functionally characterized inactive chitinase-like proteins: the *Drosophila melanogaster* imaginal disc growth factor (IDGF) and a *Mamestra brassicae* polypeptide growth factor (Fig 3). The fruit fly IDGF was first identified from the conditioned medium of *Drosophila* imaginal disc C1.8+ cells and was shown to stimulate the growth of cultured imaginal disk cells [37]. Similarly, the *M*. *brassicae* chitinase-like protein, called MbIDGF, stimulated the cell growth of two cell lines that were derived from the fat body and hemocytes of *M*. *brassicae*, in a dose-dependent manner [38].

Given the high degree of similarity (up to 70% at the amino acid level) between SPX6 and the *Drosophila* and *Mamestra* IDGFs, the probability that SPX6 also possesses the ability to promote cell growth and development is high. The discovery of the presence of such an IDGF-like protein in the spermatophylax could have important consequences with respect to female reproductive output, depending on what the target tissue of SPX6 in the female body is. To our knowledge, only a single study to date has tackled the question of where spermatophylax material ends up in the female body after it is ingested [39]. In this study, males of the katydid *Requena verticalis* were fed radioactive protein hydrolysate in order to metabolically label their spermatophores. These males were mated to virgin females that were subsequently dissected either 3 days or 9–13 days post-mating, and radioactivity was recorded in several tissues. Radioactivity measurements showed that ovaries and immature eggs of females at 3 days post-mating had higher concentrations of spermatophylax-derived proteins than did somatic tissues, but such a difference was not observed for females at 9–13 days post-mating. However, older females had developed mature (unfertilized) eggs which did harbor higher concentrations of the label [39]. If a protein such as SPX6 were to reach the ovaries intact after being ingested by a *G*. *sigillatus* female and end up in a location where it could exert its potential ability to promote cell growth and development in immature eggs, this too could represent another avenue by which males manipulate females. Although consumption of a spermatophylax by female *G*. *sigillatus* has been found to have no effect on the number of eggs produced [9, 40], it has been found to increase the rate of oviposition [40]. Such an effect would be highly advantageous to the male because female *G*. *sigillatus* are highly polyandrous, behavior that promotes a high degree of sperm competition leading to a dilution of male paternity [41]. Thus, even a transitory increase in oviposition rate would result in a greater number of eggs fertilized by a male before the female remates with another male.

### Two highly abundant carbonic anhydrase isoforms in *G*. *sigillatus* spermatophylaxes

**Fig 4:**
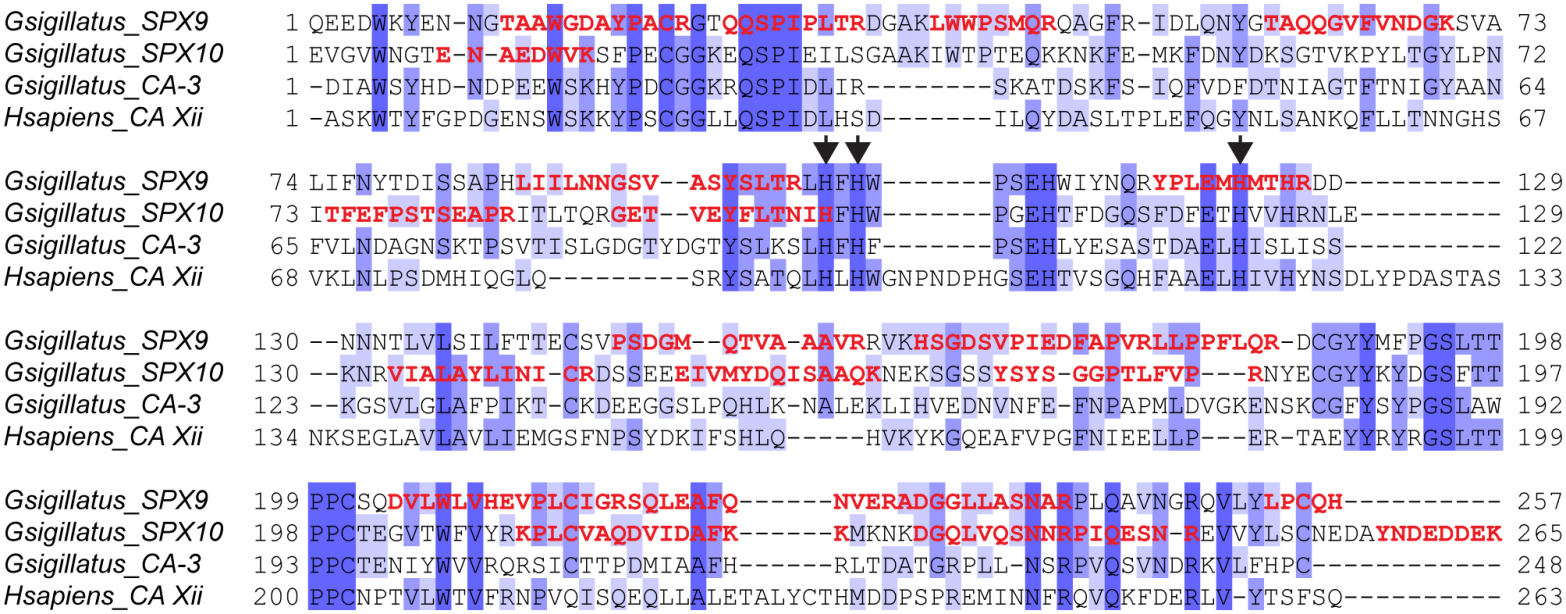
Amino acid alignment of *Gryllodes sigillatus* carbonic anhydrases (SPX9, SPX10 and CA3) found in the *G*. *sigillatus* male accessory gland transcriptome with human carbonic anhydrase Xii (CAXii) for which a crystal structure is available. Protein sequences were aligned after part of the sequence corresponding to the amino-terminal signal peptide was removed using Muscle v3.7. The degree of amino acid identity of each residue is represented by dark (strictly identical) to light purple shadings (low identity). Histidine–zinc ligands, according to the human CAXii, are indicated by arrowheads. The *de novo* sequenced peptides obtained by MS/MS which allowed the identification of SPX9 and SPX10 are indicated in red.

Two highly abundant proteins, SPX9 and SPX10, shared high similarity with other insect-derived alpha-class carbonic anhydrases (α-CAs) (Fig 1 and Table 3). CAs are ubiquitous zinc-containing metalloenzymes that catalyze the reversible hydration of carbon dioxide into a bicarbonate anion and a proton. The reaction catalyzed by CAs is crucial for the regulation of acid-base balance in organisms, but CAs have been found to participate in other physiological processes such as bone resorption in vertebrates, gluconeogenesis, the production of body fluids, and the transport of CO_2_ and HCO_3_^-^ [42]. SPX9 and SPX10 possess the same primary structure composed of an amino-terminal signal peptide and a carbonic anhydrase catalytic core. No predicted glycosylphosphatidylinositol (GPI) anchor signal could be detected, indicating that SPX9 and SPX10 are soluble extracellular proteins. In addition, we believe that these two *G*. *sigillatus* α-CAs are most likely active enzymes, because the three histidine residues implicated in the binding of the Zinc cation are conserved when compared to the human CA XII for which a crystal structure is available (Fig 4). A third carbonic anhydrase cDNA (CA3) was found in the *G*. *sigillatus* male accessory gland transcriptome (Fig 4), but the corresponding protein is likely absent from spermatophylaxes because not a single *de novo* peptide matched to CA3.

The presence of potentially active α-CAs in *G*. *sigillatus* spermatophylaxes is puzzling and the potential function of these two enzymes is difficult to understand. Do these enzymes keep the acid-base balance of the spermatophylax under control? If yes, a single α-CA isozyme would seem to be sufficient. On the other hand, what could be the impact on gut homeostasis of the release of these two α-CA isozymes after the female ingests a spermatophylax, especially related to pH stability? The pH of the gut lumen is generally well regulated but can vary within individuals mainly due to effects of temperature, activity, discontinuous ventilation, and diet [43]. In addition, the gut is highly metabolically active, and the release of carbon dioxide into the lumen is to be expected [44]. The pH of the gut luminal content in *G*. *sigillatus* varies from slightly acidic in the crop (5.0) to neutral in the gastric caeca and anterior ventriculus (6.4 to 7.3) to slightly alkaline in the posterior ventriculus and hindgut (7.8 to 8.0) [36]. In order to work efficiently, the pH optima of luminal digestive enzymes should logically correlate with the pH of the gut lumen. As a consequence, acidification or alkalinization of the gut lumen environment could dramatically impact the digestive process by decreasing the efficiency of digestive enzymes. The release into the gut lumen of the two α-CA isozymes from an ingested spermatophylax could affect the gut luminal pH and thus negatively impact the digestive capabilities of a *G*. *sigillatus* female. On top of the presence of the pacifastin serine protease inhibitor (SPX4), modifying the gut luminal pH by the action of both α-CAs (SPX9 and SPX10) would represent another way to “protect” spermatophylax proteins from proteolysis by the female’s digestive enzymes.

### Other SPX proteins with similarity to known insect proteins

SPX3 shared a high degree of similarity with members of the odorant binding protein (OBP) family (Table 3). OBPs are characterized by a highly conserved pattern of six cysteine residues forming three disulfide bridges which could also be found in SPX3. Vertebrate OBPs, which belong to the superfamily of lipocalins, present a folding pattern composed of eight antiparallel β-sheets and a short α-helical segment close to the carboxyl-terminus; in contrast, insect OBPs present a folding pattern composed of six α-helical domains arranged in a very compact structure which encloses a hydrophobic cavity. Both types of architectures are extremely stable and do not react to temperature and proteolytic digestion [45]. Insect OBPs are mostly expressed in sensory organs where they play a role in olfaction and chemoreception by detecting pheromones and other odorant molecules, but some insect OBPs have also been identified in pheromone glands and in reproductive organs, often associated with pheromone molecules [45]. Insect OBPs usually bind and carry small hydrophobic molecules such as pheromones, hormones and other semiochemicals. Accordingly, the putative function of SPX3 in *G*. *sigillatus* spermatophylaxes could be to deliver such a chemical to a target tissue in the female body. Alternatively, such an OBP may be responsible for the transport of a volatile compound (odour) which could make the spermatophylax more appealing to eat by the female recipient. Many of the free amino acids contained in the spermatophylax of *G*. *sigillatus* are known to be insect phagostimulants [8, 10] and this gift is accepted at a high rate in female *G*. *sigillatus*, as well as a number of non-gift-giving species of cricket (*Acheta domesticus*, *Gryllus veletis* and *Gryllus integer*) [13, 46]. Although it is likely that this initial appeal of the spermatophylax is linked to odours, these have not yet been examined in this species.

The presence of a protease, a serine carboxypeptidase (SPX7), in *G*. *sigillatus* spermatophylaxes is puzzling (Fig 1 and Table 3). Sequence analyses revealed that SPX7 belongs to the S10 family of serine proteases (MEROPS Accession MER002010) [47]. Carboxypeptidases usually hydrolyze single amino acids from the carboxyl-terminus of a peptide chain and can be divided into three categories according to their catalytic mechanism: Serine carboxypeptidase, metallo-carboxypeptidase and cysteine carboxypeptidase. Serine carboxypeptidases possess a reactive serine in their active site and are mostly active at acidic pH levels. The residues of the catalytic triad in family S10 occur in the order serine, aspartic acid and histidine [48]. The amino acid sequence we managed to obtain from the *G*. *sigillatus* accessory gland transcriptome was truncated at its amino terminus and contained only the region including the second and third residue of the catalytic triad (S3 Fig). Interestingly, in SPX7, the third residue of the catalytic triad, a histidine in all characterized serine carboxypeptidases [48], was replaced by a glutamine residue, indicating that SPX7 may not be an active enzyme.

SPX12 is a low-abundant protein characterized by the presence of at least four fasciclin-like (FAS1) domains (Fig 1 and Table 3). We did not manage to get the full-length cDNA sequence for SPX12 and so were missing the beginning of the sequence. SPX12 shared a high degree of similarity to homologous proteins in other insects, harboring four FAS1 domains, including the *D*. *melanogaster* midline fasciclin protein. In flies, midline fasciclin was shown to be expressed within cell bodies of midline neurons and glia, and on midline axons [49]. So far, we have found no evidence that fasciclin plays a role in insect reproductive tissues in the literature, except for the fact that some accessory gland ESTs derived from several cricket species share up to 96% amino acid identity with SPX12 (Table 4); this high degree of similarity indicates that SPX12 and its homologs may be specifically expressed in male accessory glands and may play a role in the reproductive process [19, 50].

## Conclusions

What does our study bring to the understanding of the function of spermatophylaxes in *G*. *sigillatus*? First, spermatophylaxes contain proteins, some of which are similar to known insect proteins and some of which—thanks to our proteomics analysis—are novel. Second, due to the presence of a protease inhibitor targeting trypsins and chymotrypsins, these proteins seem valuable enough to merit “protection” from proteolysis by proteases present in the digestive tract of female crickets. Third, one of the SPX proteins shows a high degree of similarity to known polypeptide growth factors described in other insects and could promote cell growth and development in its target tissue within the female’s body or possibly influence female reproduction. Our analysis of the composition of the *G*. *sigillatus* spermatophylax proteome opens up an exciting new research angle in our understanding of the evolution of nuptial food gifts in insects, mating inducements that are pervasive across insect mating systems.

## Supporting Information

S1 Fig Nucleotide alignment of transcripts encoding SPX1 and SPX2 variants. Sequences were aligned using MAFFT v7.0 and identical nucleotides were shaded in dark purple whereas less conserved nucleotides were marked with lighter shades of purple. For each sequence, the initial methionine codon (ATG) is labelled in red and the stop codon is labelled in pink.
(TIF)

S2 Fig Amino acid alignment of *G*. *sigillatus* SPX4 with a two-domain pacifastin protease inhibitor from the desert locust *Schistocerca gregaria*. Protein sequences were aligned after removing part of the sequence corresponding to the amino-terminal signal peptide using Muscle v3.7 and the degree of amino acid identity of each residue is represented by dark (strictly identical) to light purple shadings (low identity). The conserved three disulfide bridges per pacifastin domain are indicated on top of the alignment with black bars. The dibasic cleavage site between the two pacifastin domains of the desert locust protein is indicated by an arrow. For each pacifastin domain of SPX4, the “P1” residues determining the target proteases of the inhibitor domain are labelled in orange. The unique *de novo*-sequenced peptide that served for the identification of SPX4 is labelled in red.
(TIF)

S3 Fig Amino acid alignment of *G*. *sigillatus* SPX7 with other insect-derived serine carboxypeptidases from the S10 serine protease family. Protein sequences were aligned after removing part of the sequence corresponding to the amino-terminal signal peptide using Muscle v3.7 and the degree of amino acid identity of each residue is represented by dark (strictly identical) to light purple shadings (low identity). The amino acid residues corresponding to the catalytic triad of serine carboxypeptidases from the S10 family of serine proteases according to the MEROPS database are indicated by arrowheads. The *de novo*-sequenced peptides obtained by MS/MS which allowed the identification of SPX7 are indicated in red.
(TIF)

S1 File Details of the peptides used for identification of the SPX proteins against orthopteran-derived ESTs on InsectaCentral. (XLSX)

S2 File Details of the peptides used for identification of the SPX proteins against the *G*. *sigillatus* accessory gland transcriptome. (XLSX)
