# Supplementary figures and images for: What’s in the Gift? Towards a Molecular Dissection of Nuptial Feeding in a Cricket
Source: PLoS One. 2015 Oct 6;10(10):e0140191. doi: 10.1371/journal.pone.0140191 (PMC4595131; doi:10.1371/journal.pone.0140191)

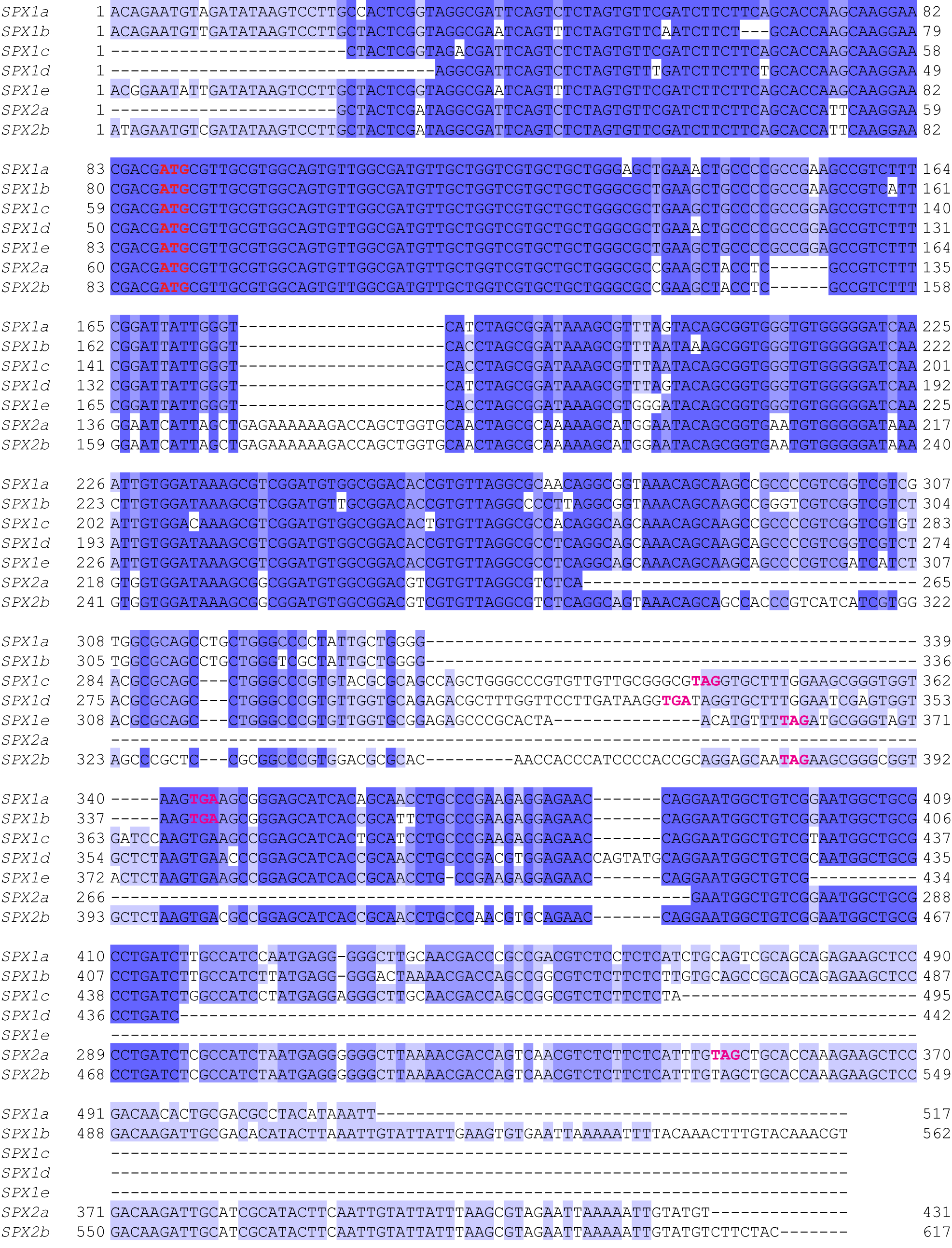

Supplement: S1 Fig — Sequences were aligned using MAFFT v7.0 and identical nucleotides were shaded in dark purple whereas less conserved nucleotides were marked with lighter shades of purple. For each sequence, the initial methionine codon (ATG) is labelled in red and the stop codon is labelled in pink. (TIF) [file pone.0140191.s001.tif]

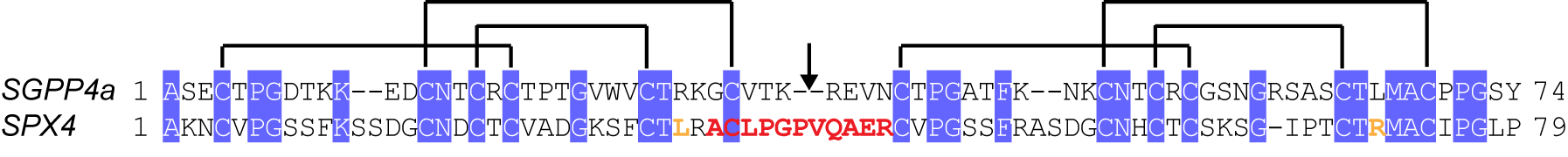

Supplement: S2 Fig — Protein sequences were aligned after removing part of the sequence corresponding to the amino-terminal signal peptide using Muscle v3.7 and the degree of amino acid identity of each residue is represented by dark (strictly identical) to light purple shadings (low identity). The conserved three disulfide bridges per pacifastin domain are indicated on top of the alignment with black bars. The dibasic cleavage site between the two pacifastin domains of the desert locust protein is indicated by an arrow. For each pacifastin domain of SPX4, the “P1” residues determining the target proteases of the inhibitor domain are labelled in orange. The unique de novo-sequenced peptide that served for the identification of SPX4 is labelled in red. (TIF) [file pone.0140191.s002.tif]

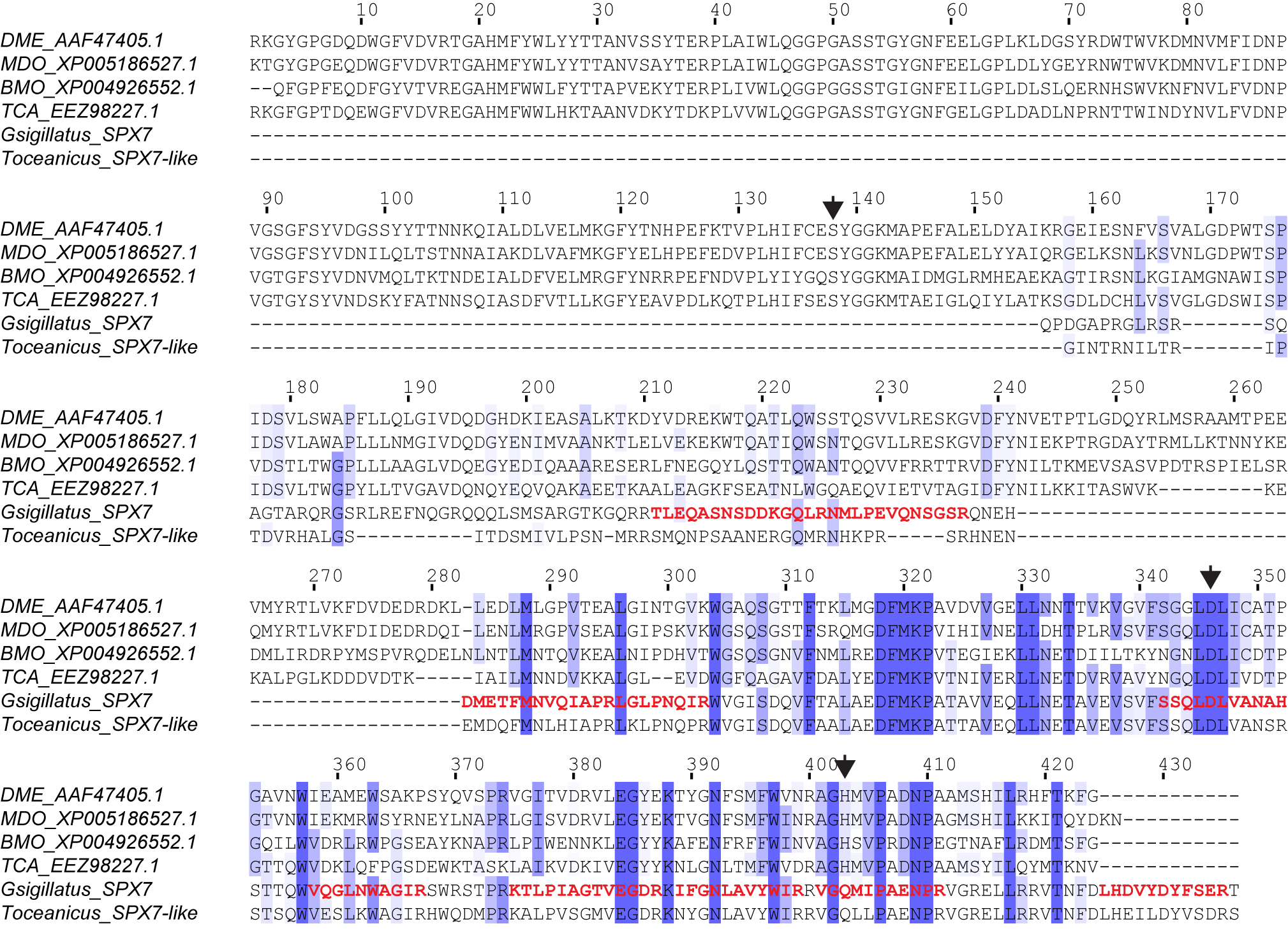

Supplement: S3 Fig — Protein sequences were aligned after removing part of the sequence corresponding to the amino-terminal signal peptide using Muscle v3.7 and the degree of amino acid identity of each residue is represented by dark (strictly identical) to light purple shadings (low identity). The amino acid residues corresponding to the catalytic triad of serine carboxypeptidases from the S10 family of serine proteases according to the MEROPS database are indicated by arrowheads. The de novo-sequenced peptides obtained by MS/MS which allowed the identification of SPX7 are indicated in red. (TIF) [file pone.0140191.s003.tif]
